# Phosphorylation Regulates CAP1 (Cyclase-Associated Protein 1) Functions in the Motility and Invasion of Pancreatic Cancer Cells

**DOI:** 10.1038/s41598-019-41346-3

**Published:** 2019-03-20

**Authors:** Huhehasi Wu, Rokib Hasan, Haitao Zhang, Joshua Gray, Dominic Williams, Morgan Miller, Faith Allen, Virlan Lee, Thomas Kelly, Guo-Lei Zhou

**Affiliations:** 1 Department of Biological Sciences, Arkansas State University, State University, USA; 2 Molecular Biosciences Graduate Program, Arkansas State University, State University, USA; 3 Department of Pathology, University of Arkansas for Medical Sciences, Little Rock, USA; 4 Winthrop P. Rockefeller Cancer Institute, University of Arkansas for Medical Sciences, Little Rock, USA

## Abstract

Pancreatic cancer has the worst prognosis among major malignancies, largely due to its highly invasive property and difficulty in early detection. Mechanistic insights into cancerous transformation and especially metastatic progression are imperative for developing novel treatment strategies. The actin-regulating protein CAP1 is implicated in human cancers, while the role still remains elusive. In this study, we investigated roles for CAP1 and its phosphor-regulation in pancreatic cancer cells. No evidence supports remarkable up-regulation of CAP1 in the panel of cancer cell lines examined. However, knockdown of CAP1 in cancer cells led to enhanced stress fibers, reduced cell motility and invasion into Matrigel. Phosphorylation of CAP1 at the S308/S310 tandem regulatory site was elevated in cancer cells, consistent with hyper-activated GSK3 reported in pancreatic cancer. Inhibition of GSK3, a kinase for S310, reduced cell motility and invasion. Moreover, phosphor mutants had defects in alleviating actin stress fibers and rescuing the reduced invasiveness in the CAP1-knockdown PANC-1 cells. These results suggest a required role for transient phosphorylation for CAP1 function in controlling cancer cell invasiveness. Depletion of CAP1 also reduced FAK activity and cell adhesion, but did not cause significant alterations in ERK or cell proliferation. CAP1 likely regulates cancer cell invasiveness through effects on both actin filament turnover and cell adhesion. Finally, the growth factor PDGF induced CAP1 dephosphorylation, suggesting CAP1 may mediate extracellular signals to control cancer cell invasiveness. These findings may ultimately help develop strategies targeting CAP1 or its regulatory signals for controlling the invasive cycle of the disease.

## Introduction

Cancer metastasis, or spreading of cancer to other parts of the body, accounts for the death of most of cancer patients, because it damages critical organs and typically eliminates surgical resection as the otherwise most effective treatment option. Morphological transformation, characterized by an aberrant actin cytoskeleton, stimulates motility and invasion of cancer cells and ultimately leads to cancer metastasis; along with the proliferative transformation, it is one of the two arguably most prominent hallmarks of cancer^1^. Largely due to its highly invasive property as well as difficulty in early detection^2^, pancreatic cancer has the worst prognosis among major cancers, with a 5-year survival rate at a mere ~4%. Given the lack of effective treatment options for this dreadful disease, insights into the mechanisms underlying cancerous transformation and especially metastatic progression are in urgent need in order to develop novel strategies for early detection and targeted therapeutics that may achieve better treatment outcomes.

Dynamic actin cytoskeletal rearrangement, based on repeated cycles of actin filament turnover, is the primary driving force of cell migration and cancer cell invasiveness^3,4^. CAP (Cyclase-Associated Protein), first identified in budding yeast^5,6^, is conserved as an actin-regulating protein across all eukaryotes tested^7,8^. Whereas its function in binding and sequestering actin monomers was initially thought to be solely responsible for its function in regulating the actin cytoskeleton, subsequent studies have revealed far more versatile roles for the protein in facilitating all key steps in the cycle of actin filament turnover, through multiple mechanisms carried out by all three of its structural domains^7,9^. Mammalian CAP1, the ubiquitously expressed isoform out of two^10^, has been more intensively studied and better understood. Work in our group and others have established roles for mammalian CAP1 in regulating the actin cytoskeleton and cell migration, including our identification of a novel function in cell adhesion^9,11–13^. Unsurprisingly, evidence is accumulating that implicates CAP1 in the invasiveness of a growing list of human cancers that include breast, pancreatic, liver, and lung cancer, and oral squamous cell carcinoma^14–19^. However, the role for CAP1 in human cancers still remains elusive, with mounting evidence that suggests a role that is dependent on the type or even subtype of cancer, where potential activation of cell adhesion signaling likely plays a key role^11,12,18^.

Considering the key function of CAP1 in facilitating cofilin-driven actin dynamics, it was speculated that up-regulation of CAP1 in cancer cells would stimulate cell invasiveness by speeding up the rate of actin filament turnover. Whereas some earlier studies support this notion, lines of emerging evidence actually argues against such a clear-cut, stimulatory role for CAP1 in cancer invasiveness. Firstly, while some studies suggest that CAP1 promotes cancer cell invasiveness^14,15,17^, up-regulation of CAP1 was not found in breast cancer cells in our well-controlled recent study; moreover, to our surprise, knockdown of CAP1 in metastatic breast cancer and HeLa cells actually stimulated cell invasiveness^12,18^. Secondly, available data to date do not support a universal up-regulation of CAP1 in cancer cells or tissues either. At least a sub-population of cancer cells in pancreatic cancer tissues had no or marginal CAP1 staining^14^. In addition, no up-regulation of CAP1 was detected in breast cancer cells^18^. Furthermore, we revealed a highly dynamic regulation of CAP1 expression levels in breast cancer cells, responding to cell culture conditions including serum starvation and stimulation^18^. Finally, a public database - The Human Protein Atlas (http://www.proteinatlas.org/ENSG00000131236-CAP1/cancer) revealed remarkable up-regulation of CAP1 only in colorectal cancer, out of 20 cancer types examined. The other cancer types, including pancreatic cancer, only had low to medium CAP1 expression.

We previously identified a new function for CAP1 in regulating FAK (Focal Adhesion Kinase) and cell adhesion, which suggests CAP1 may regulate cell motility and invasion through cell adhesion signaling, in addition to its role in regulating the actin cytoskeleton^12,18^. In HeLa and metastatic breast cancer cells, depletion of CAP1 leads to activation of FAK and enhanced cell adhesion, while in non-metastatic breast cancer cells it actually reduced FAK activity and cell adhesion^12,18^. Cell adhesion also plays a critical role in cell movement, highlighted by providing the traction force essential for pulling the cell body forward^20^. Moreover, FAK has also been intimately linked to the actin cytoskeleton, and activated FAK stimulates formation of lamellipodia^21^; indeed, enhanced lamellipodia were observed in CAP1-knockdown HeLa and metastatic breast cancer cells^12,18^. Separately, FAK can regulate ERK (External signal Regulated Kinase)^22^, to potentially control cancer cell invasiveness through the ERK/Snail/E-Cadherin axis^23^. Activation of FAK caused by CAP1 depletion in HeLa and metastatic breast cancer cells is believed to have overcome the negative effect from the reduced actin filament turnover on cell motility; as a result, knockdown of CAP1 actually stimulates cell motility and invasiveness^11,12,18^. Finally, alterations in the activity of the well-documented cell proliferation regulator ERK from CAP1 knockdown led to cell proliferation phenotypes in breast cancer cells^18^; thus, CAP1 may also play a role in cell proliferation, at least in some cancer types.

If no up-regulation of CAP1 in human cancers, then de-regulated functions of the protein is likely to be important for its role in cancer cells. We previously identified the first regulatory mechanism for CAP1, through phosphorylation at the Ser307/Ser309 (S307/S309) tandem site on mouse CAP1 (equivalent to S308/S310 on human CAP1)^24^. Transient phosphorylation at this site controls alternative association and dissociation of CAP1 with partner proteins cofilin and actin, interactions essential for CAP1 to facilitate all key steps in the cycle of actin filament turnover^9^. Interestingly, GSK3 (Glycogen Synthase Kinase 3), which we identified as a kinase for S309 (S310 for human CAP1)^24^, was reported to be hyper-activated in pancreatic cancer^25^. Both the phosphor-mimetic DD (S307D/S309D) or the unphosphorylatable AA (S307A/S309A) mutant that resists regulation through transient phosphorylation exhibited defects in alleviating the enhanced stress fibers when re-expressed in the CAP1-knockdown HeLa cells^24^.

A previous study suggested that up-regulated CAP1 promotes pancreatic cancer cell motility^14^. In this study, we sought to further, and more systematically, determine roles for CAP1 and its phosphor-regulation in pancreatic cancer cells. We report here our findings that CAP1 is required for both the motility and invasion in pancreatic cancer cells. Although no remarkable up-regulation of CAP1 was detected in the four cancer cell lines tested in a panel, elevated S308/S310 phosphorylation on CAP1 was detected in cancer cells as compared to the control cells. Our findings support the notion that phosphor-regulation is of critical importance for CAP1 functions in the actin cytoskeleton and the invasiveness of pancreatic cancer cells. Disrupting the regulation of CAP1 through transient phosphorylation by GSK3 inhibition or in the form of the re-expressed phosphor mutants, compromised CAP1 functions in alleviating the enhanced stress fibers and rescuing the invasiveness of the CAP1-knockdown PANC-1 cells. Depletion of CAP1 in pancreatic cancer cells also led to reduced FAK activity but did not cause considerable alterations in ERK, which are consistent with that no significant alterations in proliferation of pancreatic cancer cells was detected. Finally, PDGF (Platelet-Derived Growth Factor) induced dephosphorylation of CAP1 at S307/S309, suggesting that CAP1 likely links extracellular signals to cancer cell invasiveness.

## Results

### No up-regulation of CAP1, but elevated S308/S310 phosphorylation, was detected in pancreatic cancer cells

**Figure 1:**
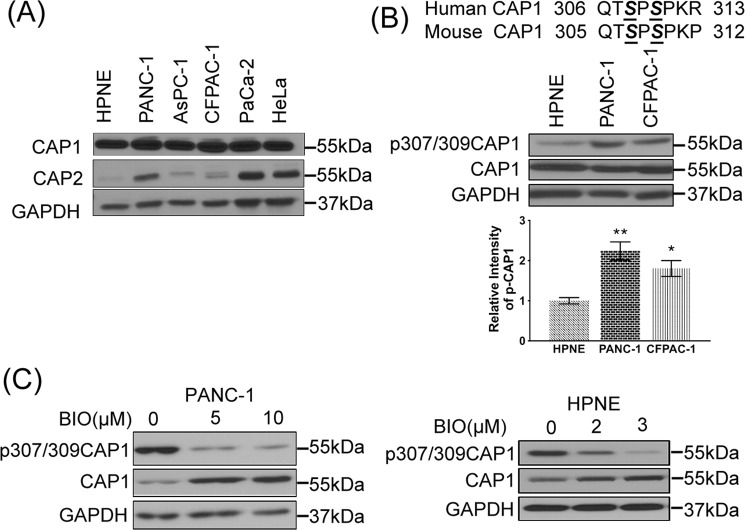
Elevated S308/S310 phosphorylation on CAP1, but not up-regulated CAP1 expression, was detected in pancreatic cancer cells. (**A**) Western blot reveals abundant expression levels of CAP1 comparable to that in the HeLa cells were detected in all four cancer cell lines, and the control pancreas cells (hTERT-HPNE) have a similar CAP1 expression level. The CAP2 blot results show that PANC-1 and Mia PaCa-2 cancer cells express abundant CAP2 levels that were remarkably higher than that in the control hTERT-HPNE cells. (**B**) Western blot reveals elevated S308/S310 phosphorylation on CAP1 in the PANC-1 and CFPAC-1 pancreatic cancer cells as compared with that in the untransformed hTERT-HPNE pancreas cells. The phosphor-specific antibody recognizes phosphor signals on both residues. The aligned sequences on top show the sequences that surround the tandem phosphor-regulatory site. The serine residues (S307 & S309 on mouse CAP1; S308 & S310 on human CAP1) in the tandem site are highlighted in the bold and italic form (also underlined). The phosphor signals were quantified using densitometry, and results from three experiments were analyzed in Student’s *t*-test and plotted in the graph where error bars represent S.E.M. “*” indicates P < 0.05 and “**” indicates P < 0.01. (**C**) Treatment of cells with GSK3 inhibitor 6-BIO for 16 hrs remarkably reduced CAP1 phosphorylation in both PANC-1 pancreatic cancer cells and the hTERT-HPNE cells, in a dose-dependent manner. 6-BIO also reduced CAP1 phosphorylation in CFPAC-1 cells similarly (not shown). GAPDH serves as a loading control in Western blotting.

CAP1 protein levels were determined in Western blotting in a panel of commonly used pancreatic cancer cell lines {PANC-1^26^, CFPAC-1^27^, AsPC-1^28^ and Mia PaCa-2^29^}, and compared to that in the immortalized but untransformed pancreas cell line hTERT-HPNE^30^, which serves as a control. As shown in Fig. 1A, all four cancer cell lines express abundant levels of CAP1 that is comparable to that in HeLa cells, which we previously reported to express abundant levels of both CAP1 and CAP2^12^. We found that the untransformed hTERT-HPNE cells expressed comparable levels of CAP1 to those in the cancer cell lines. We also examined expression levels of the other CAP isoform, CAP2, and found that PANC-1 and Mia PaCa-2 cells had considerably up-regulated CAP2 expression compared to that in the hTERT-HPNE cells (Fig. 1A).

We previously reported that GSK3 phosphorylates S309 on mouse CAP1^24^ (equivalent to S310 on human CAP1; aligned sequences shown in Fig. 1B). Given the reported hyper-activation of GSK3 in pancreatic cancer^25^, we looked into potential elevation of S308/S310 phosphorylation on CAP1 in cancer cells, using a phosphor-specific antibody that recognizes phosphor signals on both of the serine residues in Western blotting^24^. Interestingly, S308/310 phosphorylation was significantly elevated in the cancer cells compared to that in control cells (Fig. 1B, shown with quantified data for PANC-1 and CFPAC-1 cell lines only, but similar results were obtained with AsPC-1 and Mia PaCa-2 cells). Next, we inhibited GSK3 by treating cancer cells with a potent GSK3 inhibitor 6-BIO (6-bromoindirubin-3′-oxime)^31^ and found that the treatment reduced S308/S310 phosphorylation on CAP1 in PANC-1 and CFPAC-1 cells, in a dose-dependent manner (Fig. 1C, shown for PANC-1 cells only). Treatment with 6-BIO also reduced CAP1 phosphorylation in the control pancreas cells. Consistently, a more selective GSK3 inhibitor, LiCl^32^, also reduced CAP1 phosphorylation in PANC-1 and AsPC-1 cells, as shown later. These results support that GSK3 is also part of the phosphorylation machinery for CAP1 at S308/S310 in pancreatic cancer cells. It is also noted that treatment of cancer cells and control cells with 6-BIO consistently led to noticeable up-regulation of CAP1, through an unknown mechanism.

### Knockdown of CAP1 led to enhanced stress fibers as well as reduced motility and invasion of cancer cells

**Figure 2:**
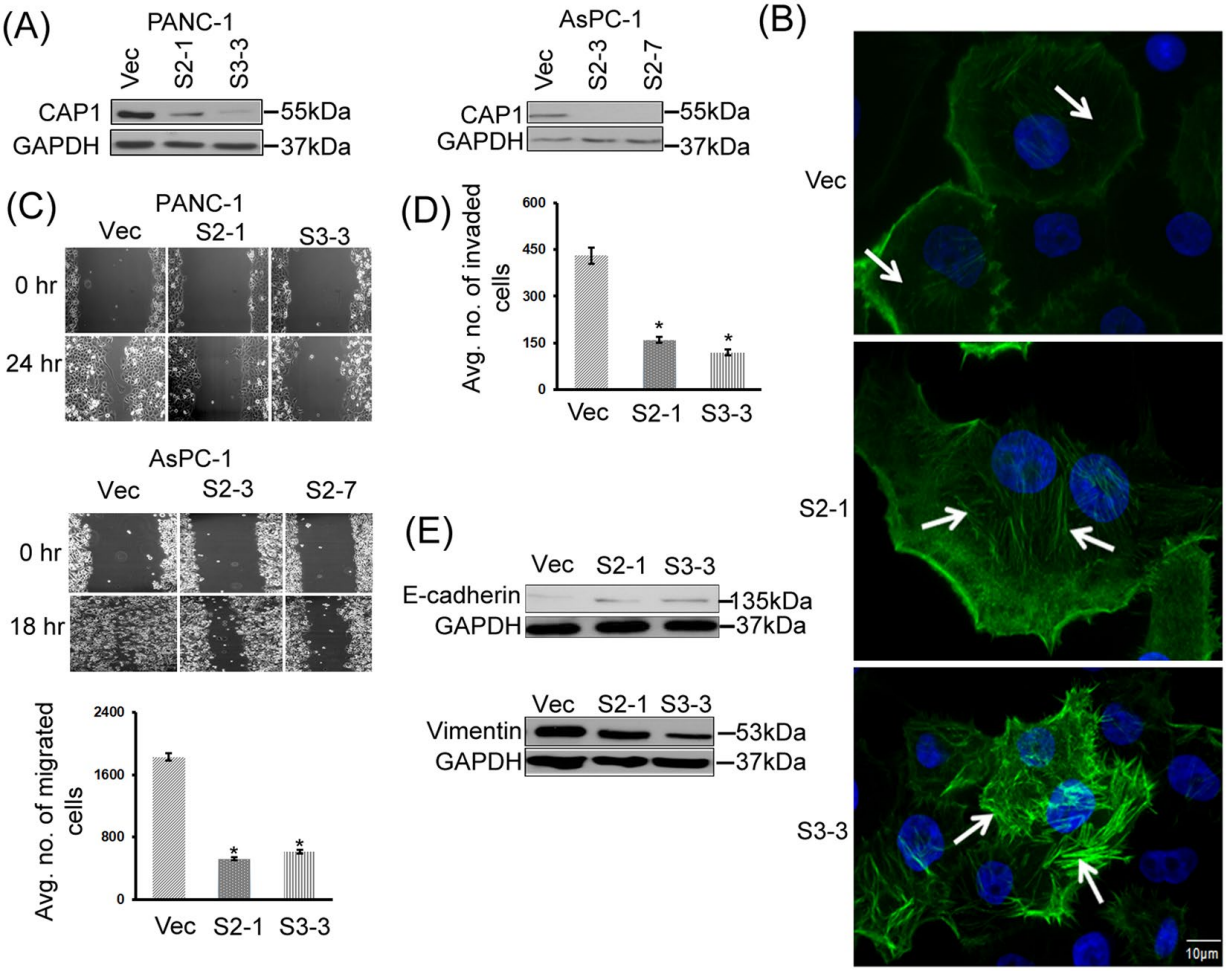
Knockdown of CAP1 in cancer cells led to enhanced actin stress fibers, and reduced cell motility and invasion. (**A**) Efficient CAP1 knockdown in two stable clones each for both PANC-1 and AsPC-1 cells as confirmed in Western blotting. CAP1 was detected in Western blotting, where GAPDH was used as a loading control. (**B**) Depletion of CAP1 in PANC-1 cells led to enhanced actin stress fibers. In control cells that harbor the empty vector for the shRNA (Vec), there were very limited actin stress fibers (indicated with arrows). In contrast, in the CAP1-knockdown cells (S2-1 and S3-3), actin stress fibers were enhanced (indicated with arrows) as observed in fluorescent microscopy following Phalloidin staining. (**C**) Depletion of CAP1 reduced motility in both PANC-1 and AsPC-1 cells as detected in the wound healing and Transwell migration assays (for PANC-1 only). For Transwell migration assays, ~2 × 10^4^ cells serum-starved overnight were loaded onto each insert placed in a well loaded with medium containing serum. After 16 hrs, migrated cells were scored and data from three independent experiments were collected, analyzed using Student’s *t*-test, and plotted in the graph where error bars represent S.E.M. “*” indicates P < 0.05 as compared to that in the control cells harboring an empty vector (Vec). (**D**) Depletion of CAP1 reduced Matrigel invasion in PANC-1 cells. The assays and data collection and analyses were conducted similarly to those in the Transwell migration assays, except that the insert membranes were pre-coated with Matrigel. “*” indicates P < 0.05 as compared with that of the control cells. (**E**) Reduced EMT in the CAP1 knockdown PANC-1 cells, as indicated by increased E-Cadherin expression as well as reduced Vimentin expression levels in the CAP1-knockdown stable clones. GAPDH serves as a loading control in Western blots.

We next attempted to silence CAP1 to determine the roles of CAP1 in pancreatic cancer cells. The stable knockdown paradigm we previously developed is compatible with a rescue strategy, which allows verification of specificity of derived phenotypes from depletion of CAP1^12,18,24^. Using two shRNA constructs S2 and S3 that target independent nucleotide sequences and effectively silenced CAP1 in HeLa and breast cancer cells^12,18,24,33^, we were able to generate stable clones with efficient CAP1 knockdown in the PANC-1 and AsPC-1 cells, as confirmed in Western blotting (Fig. 2A). Two stable knockdown clones each derived from PANC-1 (S2-1 and S3-3) and AsPC-1 (S2-3 and S2-7), were established respectively through Neomycin selection. Notably, attempts to silence CAP1 in CFPAC-1 and Mia PaCa-2 cancer cell lines, however, were unsuccessful. The CFPAC-1 cells failed to form colonies following antibiotic selection, whereas none of the approximately two dozen stable colonies screened had substantial reduction of CAP1 expression in Mia PaCa-2 cells (data not shown).

We first examined morphological and actin cytoskeletal alterations in the CAP1-knockdown PANC-1 and AsPC-1 cells. Unlike the increased cell size in the CAP1-knockdown HeLa and metastatic breast cancer cells^12,18,24^, depletion of CAP1 did not cause noticeable increase in the size of PANC-1 or AsPC-1 cells. We next stained the actin cytoskeleton in the PANC-1 cells with Phalloidin, followed by fluorescent microscopy. PANC-1 cells (and pancreatic cancer cells in general) appear to have poorly organized actin cytoskeleton structures, especially in terms of the stress fibers (Fig. 2B), in relative to the cell lines commonly used for actin cytoskeleton studies, such as HeLa and NIH3T3 fibroblasts^12,24^. Nevertheless, enhanced stress fibers were evident in the CAP1-knockdown PANC-1 cells as compared to that in the control cells (Fig. 2B). All 26 control cells that harbor an empty vector examined showed very limited presence of stress fibers. In contrast, 20 out of 27 (74.1%) of S2-1 and 18 out of 23 (78.3%) of S3-3 CAP1-knockdown cells examined had remarkably enahnced stress fibers, simlar to that shown in Fig. 2B. Accumulation of stress fibers has consistently been observed in other cells with CAP1 depletion^12,13^, a phenotype that is believed to have derived from the loss of both CAP1 functions in sequestering actin monomers and in promoting actin filament turnover.

Consistent with the enhanced stress fibers, depletion of CAP1 has been reported to reduce motility in a number of mammalian cell types, including cancer cells^13,14,17–19^. Transient knockdown of CAP1 in pancreatic cancer cells was also found to reduce cell motility in wound healing assays^14^. We tested the effect of stable CAP1 knockdown on pancreatic cancer cell invasiveness, by conducting wound healing assays, Transwell migration assays as well as Matrigel invasion assays. Depletion of CAP1 substantially reduced cell motility in the wound healing assays for both PANC-1 and AsPC-1 cells (Fig. 2C), which is consistent with the previously reported results^14^. The wounds in the CAP1-knockdown PANC-1 cells (both S3-3 and S2-1) healed only marginally 24 hrs after the introduction, whereas in the control cells the gap had almost completely filled. Similar effects were observed in the stable CAP1-knockdown AsPC-1 clones S2-3 and S2-7 (Fig. 2C). Results from the Transwell migration assays of PANC-1 cells were consistent with those from wound healing assays, as shown in the graph of quantified results in the lower panel (Fig. 2C). Moreover, the invasion assays reveal that depletion of CAP1 also reduced the capability of PANC-1 cancer cells to penetrate and invade through Matrigel (Fig. 2D). Student’s *t*-test analyses of data collected from three independent assays reveal significantly reduced invasion in the CAP1-knockdown PANC-1 stable cells (Fig. 2D). Finally, since EMT (Epithelial-Mesenchymal Transition) is related to cancer cell invasiveness, we tested the potential effect of CAP1 knockdown on EMT. Indeed, the CAP1-knockdown PANC-1 cells had up-regulated E-Cadherin, which indicates reduced EMT (Fig. 2E). We also tested another EMT marker, Vimentin, and found that depletion of CAP1 reduced its expression (Fig. 2E). Together, these results support a required role for CAP1 in the invasiveness and EMT in the PANC-1 cancer cells.

### Inhibition of GSK3, which suppresses CAP1 phosphorylation, reduced motility and invasion of cancer cells

**Figure 3:**
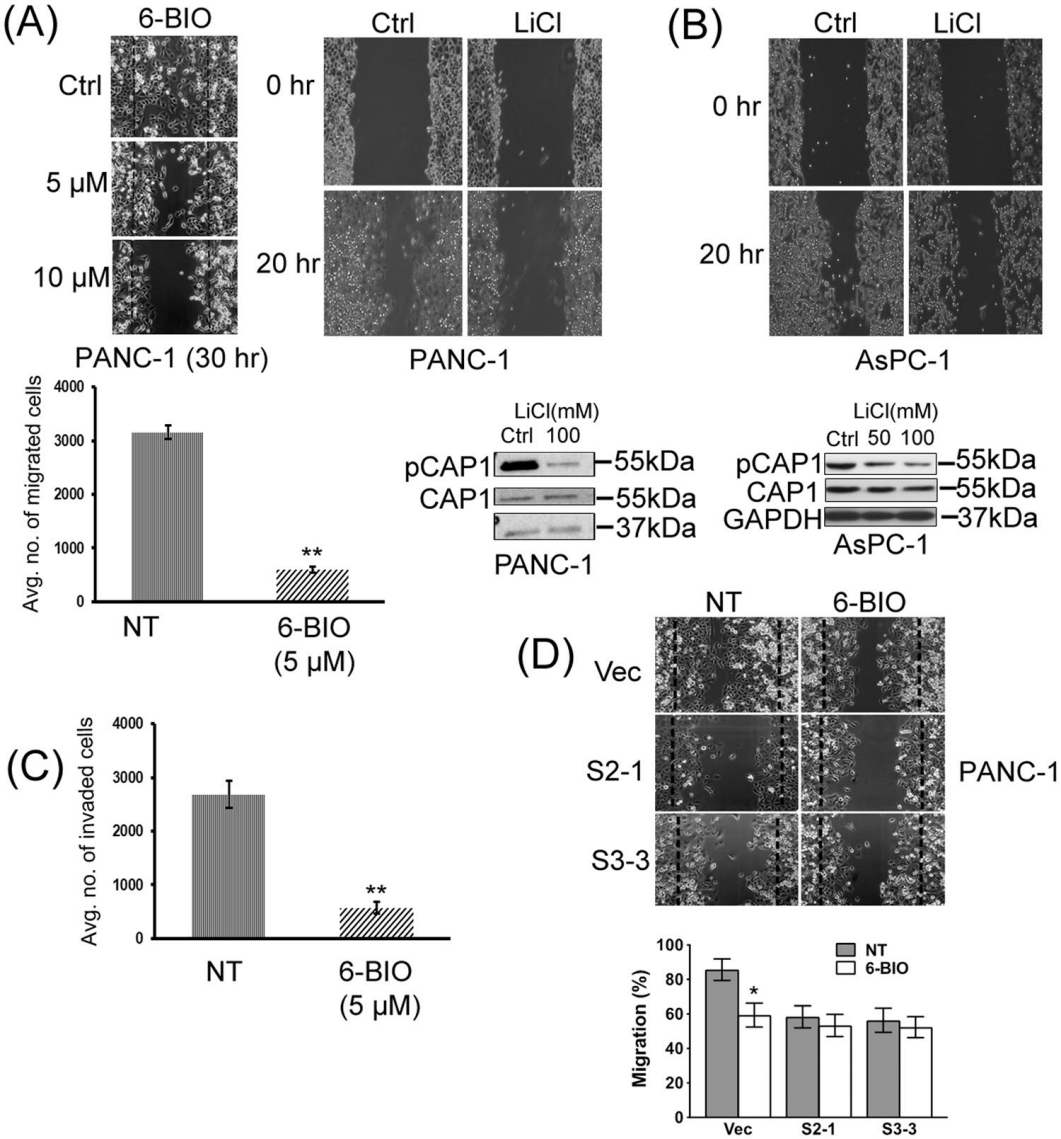
Inhibition of GSK3, which reduced CAP1 phosphorylation, also compromised cancer cell motility and invasion. (**A**) Treatment of PANC-1 cells with 6-BIO or LiCl reduced cell motility in the wound healing and Transwell migration assays. Treatment with 100 mM LiCl effectively reduced CAP1 phosphorylation in PANC-1 cells as well. Transwell migration assays with PANC-1 cells were conducted similarly as described in Fig. 2 where NT indicates cells without 6-BIO treatment. (**B**) Treatment with 100 mM LiCl also noticeably reduced CAP1 phosphorylation in AsPC-1 cells, as well as cell motility in the wound healing assays. (**C**) Treatment of PANC-1 cells with 5 μM 6-BIO significantly reduced cancer cell invasion in Matrigel invasion assays. Matrigel invasion assays, including data collection and analyses, were conducted similarly as described in Fig. 2D. (**D**) Knockdown of CAP1 in PANC-1 cells compromised the effect of 6-BIO in reducing cell motility in wound healing assays. Control and CAP1-knockdown stable PANC-1 clones were cultured for 16 hrs following the introduction of the wound, and the dashed lines indicate edges of the initial gap. Cell motility was quantified, analyzed using Student’s *t*-test, and plotted in the graph where error bars represent standard deviation. “*” indicates P < 0.05 and “**” indicates P < 0.01.

Our previous findings suggest that transient phosphorylation is crucial for the CAP1 function in regulating the actin cytoskeleton^24^. The elevated CAP1 phosphorylation in pancreatic cancer cells, consistent with the activated GSK3, suggests that phosphor-regulation may also play a role for CAP1 function in cancer cell invasiveness. We tested this possibility by inhibiting GSK3, which suppresses S308/S310 phosphorylation and meanwhile disrupts CAP1 regulation through transient phosphorylation at the regulatory site. PANC-1 cells were treated with 6-BIO followed by cell migration and invasion assays. Effects of the reduced S308/S310 phosphorylation on CAP1, by treatment with 5 μM 6-BIO (Fig. 1C), were tested first. As shown in Fig. 3A, 6-BIO treatment significantly reduced motility of PANC-1 cells in both the wound healing and Transwell migration assays. We also confirmed that treatment of PANC-1 and AsPC-1 cells with another GSK3 inhibitor, LiCl, reduced CAP1 phosphorylation as well as cell motility in wound healing assays (Fig. 3A,B). Furthermore, 6-BIO treatment also reduced Matrigel invasion of PANC-1 cells (Fig. 3C). Finally, since GSK3 is known to regulate a wide range of cell functions through a plethora of substrate molecules, the effect of GSK3 inhibition on cell motility is likely to be a collective output through multiple GSK3 targets that are involved in the regulation of the cytoskeleton, cell polarization and migration^34,35^. We thus tested and compared the effects of 6-BIO in reducing cell motility in the CAP1-knockdown PANC-1 cells and control cells. As shown in the graph in Fig. 3D, 6-BIO treatment significantly reduced the motility of the control (Vec) cells but not that of the CAP1-knockdown (S2-1 and S3-3) cells in the wound healing assays. Taken together, these results support that phosphor-regulation through S308/S310 plays an important role for CAP1 to promote the motility and invasion in pancreatic cancer cells.

### Phosphor mutants of CAP1 had compromised functions in alleviating the enhanced stress fibers and promoting development of lamellipodia in the CAP1-knockdown PANC-1 cells

**Figure 4:**
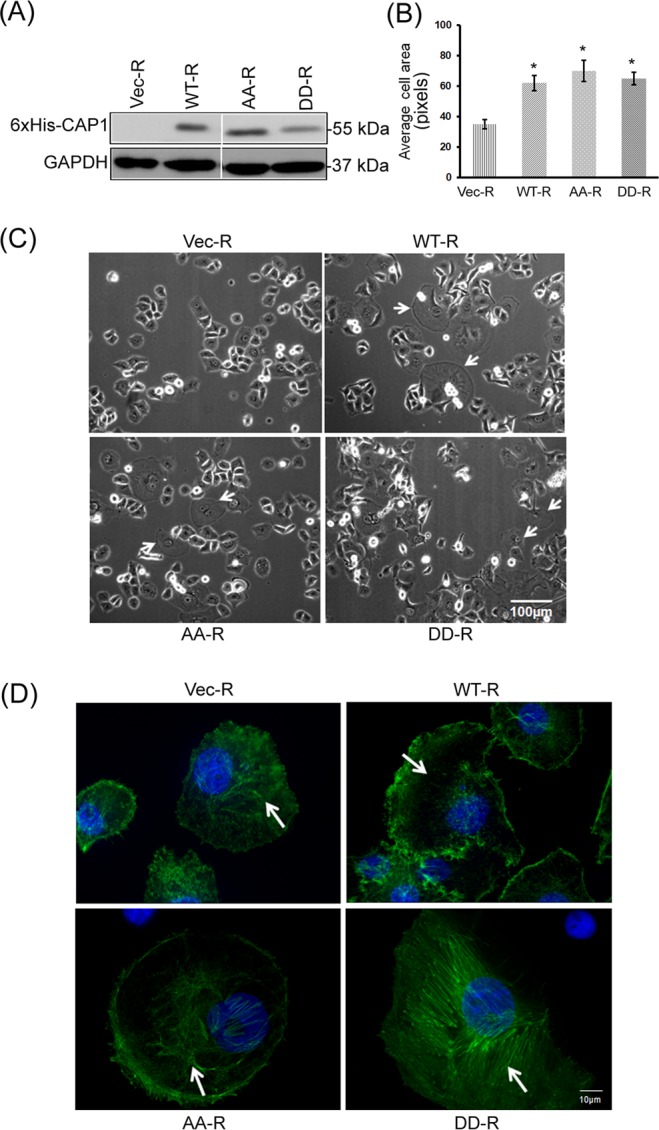
Effects of re-expression of WTCAP1 and the phosphor mutants in the CAP1-knockdown PANC-1 cells on the actin cytoskeleton, cell size and lamellipodia development. (**A**) Confirmation of re-expression of WTCAP1 and the AA and DD phosphor mutants in the S3-3 CAP1-knockdown PANC-1 stable cells in Western blotting using the antibody against the 6xHis tag. Two irrelevant lanes had been removed from the original Western blot image (shown in Supplementary Fig. 1); (**B**) quantified data showing significantly increased cell size in cells re-expressing WTCAP1 or the phosphor mutants in the CAP1-knockdown cells. Sizes of 50 cells per field were measured using the Image-J program. The data were analyzed using Student’s *t*-test, and plotted in the graph where the error bars indicate the standard deviation. “*” indicates P < 0.05 as compared to the control cells harboring the empty vector (Vec-R). (**C**) Phase images show that re-expression of WTCAP1 or the phosphor mutants stimulated the formation of lamellipodia. Some of the cells re-expressing WTCAP1 (WT-R) developed extremely large-sized lamellipodia (indicated with arrows). Re-expression of the AA (AA-R) or DD (DD-R) mutant also simulated the formation of lamellipodia, but their sizes are noticeably smaller compared to those in the cells re-expressing WTCAP1. No such lamellipodia were observed in the control CAP1-knockdown cells harboring the empty vector (Vec-R). (**D**) Re-expression of WTCAP1 in the CAP1-knockdown PANC-1 cells reduced stress fibers, whereas the cells re-expressing the mutants had enhanced stress fibers. The cells re-expressing the phosphor-mimetic DD mutant had the most enhanced stress fibers. The arrows indicate the stress fibers, or lack thereof in the case of the WT-R cells.

Our findings from the CAP1-knockdown cells support that CAP1 is required for cancer cell motility and invasion, and results from GSK3 inhibition suggest that S308/S310 phosphorylation plays a key role in the CAP1 functions. We next employed a re-expression strategy to further establish these cases. Wild type CAP1 (WTCAP1) and the phosphor mutants that mimic either phosphorylated (S307D/S309D; DD) or unphosphorylatable (S307A/S309A; AA) forms of CAP1, as described previously^12,18,24^, were stably re-expressed in the CAP1-knockdown PANC-1 cells to test their capabilities in rescuing the actin cytoskeletal and cell morphological phenotypes. These mutants harbor mismatches to the S3 shRNA target sequence to avoid recognition of the derived mRNAs by the shRNA stably present in the knockdown cells, but without altering any amino acid on CAP1^12^. We were able to establish stable clones that re-express WT mouse CAP1, or the AA and DD phosphor mutant, as confirmed in Western blotting against the 6xHis tag (Fig. 4A). Note that two irrelevant lanes had been removed from the original Western blot image (samples in those two lanes were used to confirm re-expression of two serine 36 phosphor mutants at the N-terminus). The entire set of the original Western blot result is shown in Supplementary Fig. 1. The morphology of cells re-expressing either the AA (AA-R) or DD (DD-R) mutant was examined in phase microscopy, and compared to that in the cells re-expressing WTCAP1 (WT-R). It has been reported that knockdown of CAP1 in some mammalian cell types, including HeLa and metastatic breast cancer cells, led to significantly increased cell size^12,13,18^, a phenotype we previously confirmed to be specific to knockdown of CAP1^12,18^. CAP1-knockdown in PANC-1 cells or AsPC-1 cells, however, did not show that effect. Rather, stable re-expression of WTCAP1 or the phosphor mutants in the knockdown cells actually significantly increased cell size (Fig. 4B). Moreover, re-expression of WTCAP1 led to the development of robust-sized lamellipodia (indicated with arrows), a subcellular structure that is rich in filamentous actin and is critical in driving directional cell movement (Fig. 4C), whereas virtually none of the knockdown cells harboring an empty control vector developed any such lamellipodia. Re-expressed AA or DD mutant also stimulated the formation of lamellipodia to some extent, but their sizes were noticeably smaller compared to that in the cells re-expressing WTCAP1 (indicated with arrows in Fig. 4C). We counted 200 cells for each cell type, and the percentages of cells that harbor large-sized lamellipodia were: 0% (0/200) for the knockdown cells (Vec), 14.5% (29/200) for the WT-R cells, and 9% (18/200) and 7.5% (15/200), respectively, for the AA-R and DD-R cells.

We next looked into the capabilities of the phosphor mutants in alleviating the enhanced stress fibers in the CAP1-knockdown PANC-1 cells. As shown in the confocal images in Fig. 4D, re-expression of WTCAP1 (WT-R) effectively alleviated the enhanced stress fibers, thereby rescuing the phenotype, and the stress fibers were dissolved in large areas indicated with an arrow. In contrast, the phosphor mutants were less effective in alleviating the enhanced stress fibers. Cells re-expressing the AA mutant had modestly enhanced stress fibers than the cells rescued by WTCAP1, while the cells re-expressing the DD mutant appeared to have even more enhanced stress fibers. These results are consistent with our previous findings from HeLa cells that suggest dephosphorylated CAP1 is the ‘active’ form in relative to phosphorylated CAP1^24^, while transient phosphorylation is believed to be required for optimal cellular functions of CAP1. These results also suggest that transient S308/S310 phosphorylation is important for CAP1 to regulate the actin cytoskeleton in pancreatic cancer cells; disrupting regulation through transient phosphorylation leads to defects in CAP1 function in promoting actin filament turnover.

### CAP1 phosphor mutants had defects rescuing the reduced invasiveness in the CAP1-knockdown cancer cells

**Figure 5:**
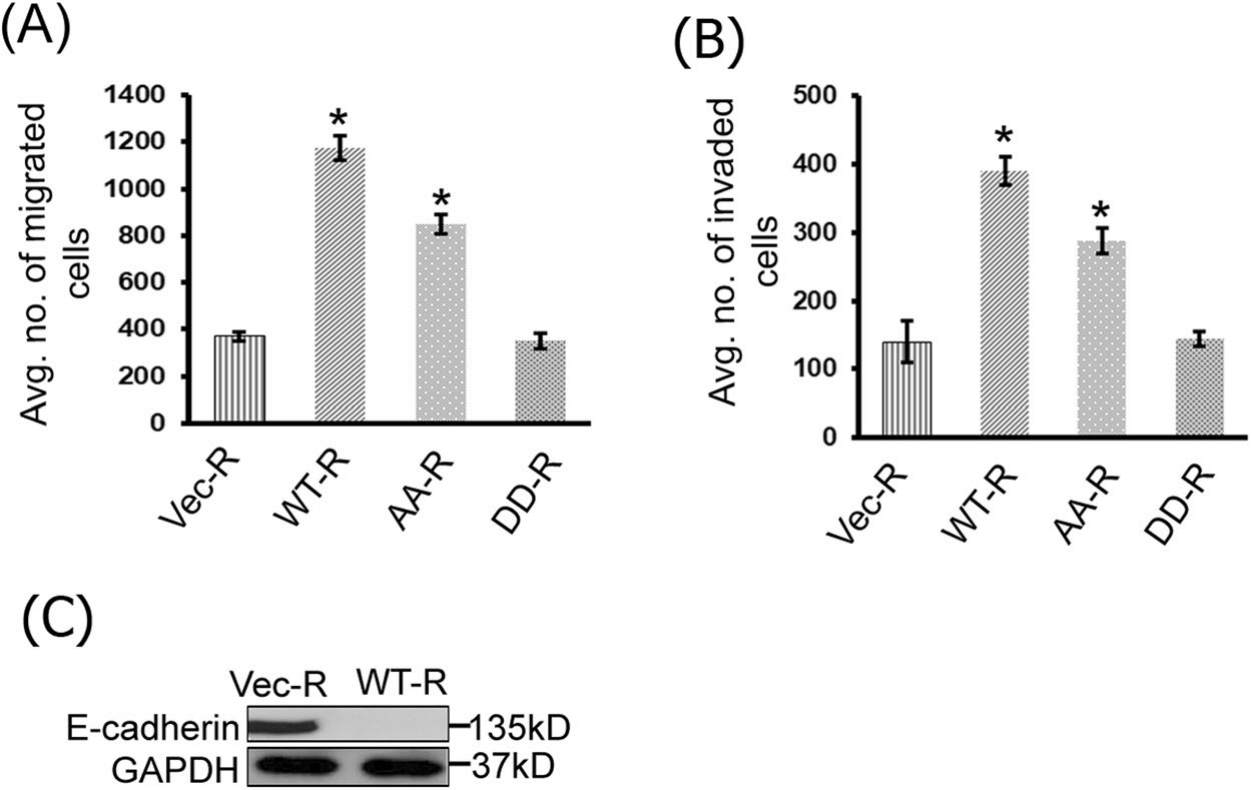
Effects of WTCAP1 and the phosphor mutants in rescuing the reduced motility and invasion in the CAP1-knockdown PANC-1 cells. (**A**) WTCAP1 and the AA mutant effectively rescued the reduced motility in the CAP1-knockdown PANC-1 cells in Transwell migration assays, while the DD mutant failed in doing so. The experiments, data collection and analyses were conducted similarly to that described in Fig. 2. The error bars represent S.E.M., and “*” indicates P < 0.05 as compared to the control cells harboring an empty vector (Vec-R). (**B**) WTCAP1 and the AA mutant effectively rescued the reduced Matrigel invasion of the CAP1-knockdown PANC-1 cells, while the DD mutant also failed to do so. The experiments, data collection and analyses were conducted similarly to that described in Fig. 2. The error bars represent S.E.M., and “*” indicates P < 0.05 as compared to the control cells. (**C**) Re-expression of WTCAP1 also rescued the up-regulated E-Cadherin in the CAP1-knockdown PANC-1 cells.

Since dynamic actin filament turnover is the primary driving force of cell movement, we next tested how well the phosphor mutants rescue the reduced cell motility and invasion in the CAP1-knockdown PANC-1 cells. We first conducted Transwell migration assays, and found that re-expression of WTCAP1 significantly increased cell motility compared to the control cells that harbor an empty vector, as shown in quantified results in the graph (Fig. 5A). The re-expressed AA mutant (AA-R), while not as effective as WTCAP1, partially rescued the reduced cell motility in the Transwell migration assays. In contrast, the DD mutant (DD-R) failed to rescue the reduced cell motility, and the rate was comparable to that in the CAP1-knockdown cells harboring an empty vector. These results are consistent with the capabilities in rescuing the enhanced stress fibers by WTCAP1 and the phosphor mutants. We further tested rescue of the reduced Matrigel invasion in the CAP1-knockdown cells, as shown in the quantified results in Fig. 5B. Similarly, WTCAP1 rescued the reduced invasion in the CAP1-knockdown PANC-1 cells most effectively; the AA mutant achieved a partial rescue while the DD mutant literally failed to rescue the phenotype. Finally, re-expression of WTCAP1 in the CAP1-knockdown cells also reduced E-Cadherin expression (Fig. 5C), further supporting that CAP1 is required for EMT in PANC-1 cancer cells. Together, these results support that phosphor-regulation through the S308/S310 tandem site plays an important role for CAP1 to promote the motility and invasion of pancreatic cancer cells.

### Evidence supporting that CAP1 mediates extracellular growth factor signals to control cancer cell invasiveness

**Figure 6:**
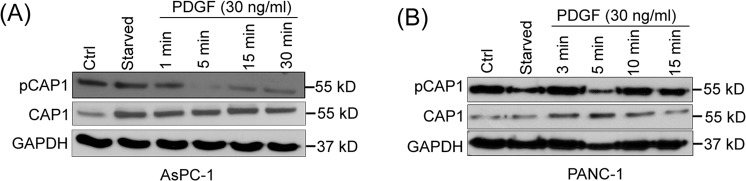
PDGF induced dephosphorylation of CAP1 in pancreatic cancer cells. (**A**) Treatment of serum-starved AsPC-1 cancer cells with PDGF induced CAP1 dephosphorylation at S308/S310. Cells were cultured overnight, serum-starved for 24 hrs, followed by treatment with 30 ng/ml PDGF for indicated time durations. Cell lysates were prepared and used in the Western blotting with a phosphor-specific antibody that detects phosphor signals on both residues of the tandem regulatory site on CAP1. The dephosphorylation effect was most significant at the 5-minute time point of PDGF treatment. (**B**) Treatment of serum-starved PANC-1 cells with PDGF also induced remarkable CAP1 dephosphorylation, in similar fashion with AsPC-1 cells. Treatment of cells and Western blotting were conducted following the same procedures as used for AsPC-1 cells. GAPDH served as a loading control in Western blot.

Physiological stimuli, such as growth factors PDGF and HGF (Hepatocyte Growth Factor)^36^, are known to stimulate rearrangement of the actin cytoskeleton and cell migration. Since phosphor-regulation at S308/S310 is crucial for CAP1 functions in regulating the actin cytoskeleton and cancer cell invasiveness, we looked into the possibility that these stimuli may regulate CAP1 phosphorylation, and through this to control the actin cytoskeletal rearrangement and cancer cell invasiveness. Interestingly, treatment of serum-starved PANC-1 and AsPC-1 cells with PDGF reduced S308/S310 phosphorylation on CAP1, most remarkably at the 5-minute time point (Fig. 6A,B). Treatment of pancreatic cancer cells with HGF or serum did not have a considerable effect in inducing the dephosphorylation of CAP1 (Supplementary Fig. 2; shown for PANC-1 cells). Therefore, signaling through CAP1 is likely at least partially responsible for PDGF to stimulate actin cytoskeletal reorganization and cancer cell invasiveness. These results are also consistent with the notion that dephosphorylated CAP1 is the ‘active’ form, as suggested by multiple lines of evidence including cofilin binding, subcellular localization of the phosphor mutants, and elevated phosphorylation of CAP1 in cells cultured in suspension^24^. However, transient phosphorylation at the tandem regulatory site, namely the cycling between the phosphorylated and dephosphorylated forms, is believed to be required for optimal cellular functions of CAP1^24^. The CAP1-dephosphorylation signals likely function in cohort with CAP1-phosphorylation signals, to regulate CAP1 cell functions through driving transient phosphorylation at S308/S310.

### Depletion of CAP1 in pancreatic cancer cells reduced FAK activity but did not cause alterations in ERK or cell proliferation

**Figure 7:**
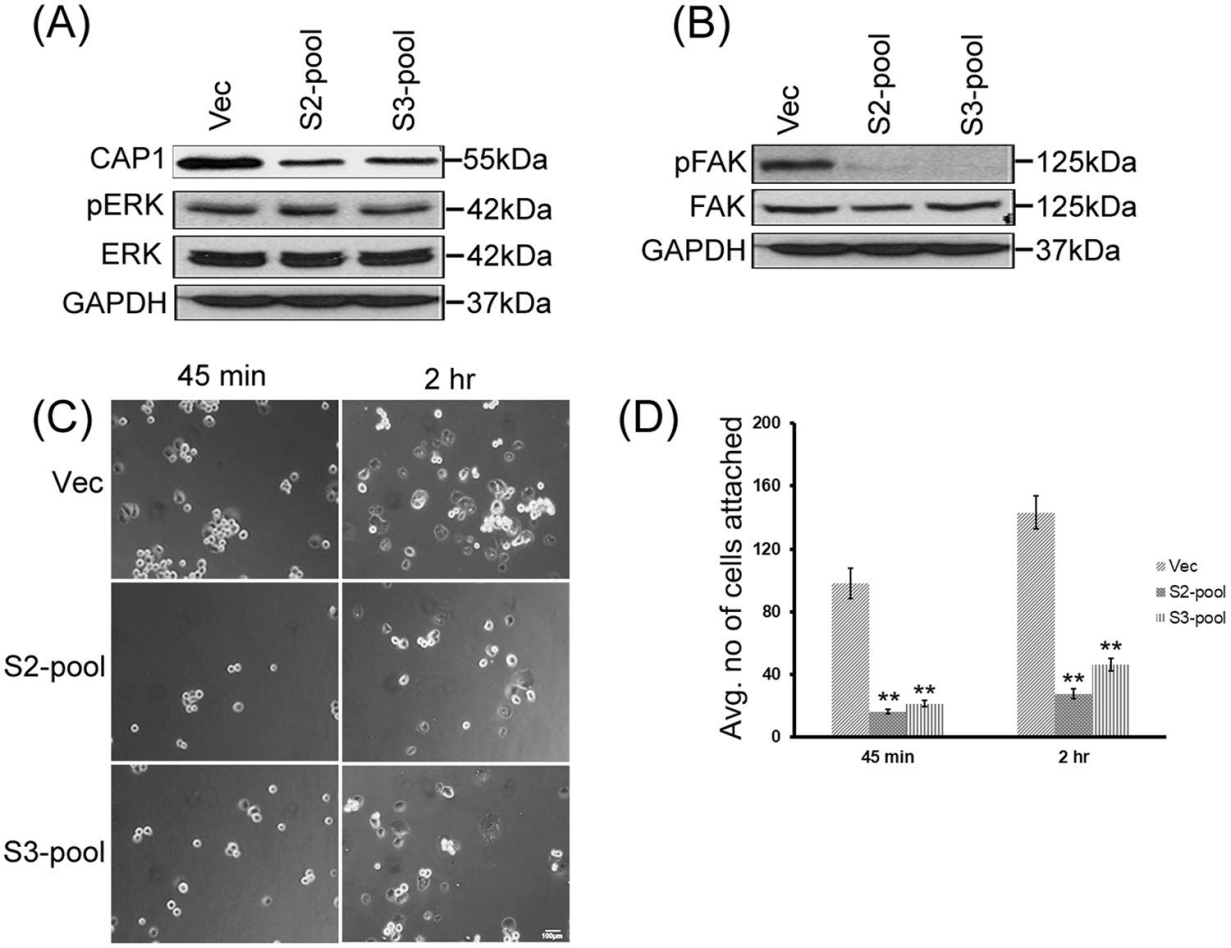
CAP1-knockdown PANC-1 pool cells had reduced FAK activity and cell adhesion, but no alterations in ERK. (**A**) CAP1-knockdown PANC-1 pool cells, derived from both S2 and S3 shRNA constructs, did not show altered ERK expression or activity compared with the control pool cells harboring an empty vector, as detected in Western blotting. ERK activity was detected in Western blotting using a phosphor-specific antibody against the Thr202/Tyr204 sites. GAPDH served as a loading control. (**B**) Reduced FAK activity was detected in the CAP1-knockdown PANC-1 pool cells as compared with the control pool cells. FAK activity was assessed by phosphorylation at Tyr397, using a phosphor-specific antibody against the site in Western blotting; (**C**) CAP1-knockdown pool cells had reduced cell adhesion at the tested time points (45 minutes and 2 hrs) following plating cells onto fibronectin-coated plates. Approximately 2 × 10^4^ cells were plated onto each well of a 6-well plate, and cells not attached at the indicated time points were washed off. The number of attached cells was counted in five random fields under a phase microscope and images taken. (**D**) Data collected from three independent cell adhesion assays were analyzed using Student’s *t*-test, and plotted on the graph where the error bars represent standard deviation. “**” Indicates P < 0.01 as compared to the control cells harboring the empty vector.

We recently identified a novel role for CAP1 in regulating the proliferation of breast cancer cells, where depletion of CAP1 exerts cell context-dependent effects on proliferation, accompanied with consistent alterations in ERK activity^18^. We tested if CAP1 may also regulate ERK and proliferation in pancreatic cancer cells. No significant alterations in the expression or phosphorylation of ERK was detected in the CAP1-knockdown PANC-1 stable clones as compared to that in the control cells (Supplementary Fig. 3A). We also conducted MTT assays testing proliferation of the S2-1 and S3-3 stable knockdown cells and comparing them to that of the control (Vec) cells, and detected slightly decreased rates of cell proliferation in the knockdown cells (Supplementary Fig. 3B). However, the differences were statically insignificant, with the P values comparing O.D. (at 570 nm) between S2-1 cells and control cells at 0.219, and that between S3-3 cells and control cells at 0.223. These results indicate that CAP1 does not play a significant role in regulating proliferation in pancreatic cancer cells. Furthermore, given that the overall tendency that stable knockdown paradigm is prone to clone variations, we generated pools of CAP1 stable knockdown PANC-1 cells, which are believed to more accurately reflect effects of CAP1 depletion on ERK, by avoiding potential influence from selection bias. As shown in Fig. 7A, pools of PANC-1 stable cells with efficient CAP1 knockdown, derived from both the S2 and S3 shRNA constructs, were established following Neomycin selection, as confirmed in Western blotting. Consistent with the findings from using stable knockdown clones, no remarkable alterations in ERK expression or its phosphorylation was detected in the knockdown pool cells as compared to the control pool cells.

Knockdown of CAP1 in breast cancer cells caused distinct alterations in FAK in the metastatic and non-metastatic breast cancer cells^18^. We also detected reduced FAK activity, without alterations in FAK expression levels, in the CAP1-knockdown PANC-1 pool cancer cells (Fig. 7B). Based on these results, we further tested effects of CAP1 depletion on cell adhesion in adhesion assays, similarly as we performed previously^12,18^. We found that the CAP1-knockdown pools cells derived from both shRNA constructs had remarkably reduced cell adhesion compared to the control cells (Fig. 7C). At both time points (45 minutes and 2 hrs) after the cells had been plated onto fibronectin-coated surface, significantly more control cells attached as compared to the CAP1-knockdown pool cells. Further, more of the attached control cells had also fully spread (developed lamellipodia and cells do not show as bright) compared to the CAP1-knockdown cells (Fig. 7C). We scored the numbers of attached cells from three fields for comparison, and data from three independent experiments were quantified as plotted in the graph (Fig. 7D).

## Discussion

Pancreatic cancer is a dreadful disease, largely due to its highly invasive property and difficulty in early detection. CAP1 is implicated in pancreatic cancer, among a number of other malignancies, while its role remains to be better established along with underlying mechanisms. This study not only further establishes the role for CAP1 in regulating the motility of pancreatic cancer cells, but also reveals the role in regulating the invasion of pancreatic cancer cells, accompanied by mechanistic insights including the phosphor-regulation of the CAP1 functions and involvement of FAK. Our findings support that phosphor-regulation through S308/S310 is crucial for CAP1 to regulate the actin cytoskeleton and promote invasiveness in pancreatic cancer cells. Cell signals that phosphorylate and dephosphorylate CAP1, such as GSK3 and those downstream of PDGF, likely function as a cohort to regulate CAP1 by facilitating the transient S308/S310 phosphorylation.

A previous study reported up-regulation of CAP1 in pancreatic cancer^14^, a conclusion drawn partially from comparison of CAP1 expression levels in cancer cells to that in normal pancreas tissue. We did not detect up-regulation of CAP1 in any of the four pancreatic cancer cell lines tested, by directly comparing the expression levels to that in the immortalized but untransformed hTERT-HPNE pancreas cells^30^. The hTERT-HPNE cell line is probably the best control available as untransformed cells, which was challenging to establish. However, we cannot exclude the possibility that the immortalized or the intermediary nature of the hTERT-HPNE cells may have caused up-regulation of CAP1 to certain extent, as compared to that in truly “normal” pancreas cells. Our results from CAP1 knockdown and re-expression reveal that CAP1 is indeed required for both the motility and invasion of pancreatic cancer cells. This is consistent with the findings from the previous study that transient knockdown of CAP1 reduced cell motility in wound healing assays^14^. Our study further establishes a required role for CAP1 in Matrigel invasion, which mimics tissue invasion in the body and is believed a better predictor of the metastatic potential of cancer cells. Importantly, we confirmed the specificity of these effects by re-expressing CAP1 in the knockdown cells to assess the rescue effects, a strategy that also allowed us to determine the functionality of the CAP1 phosphor mutants. Notably, we found that PANC-1 and Mia PaCa-2 cancer cells express up-regulated levels of CAP2 compared to that in the control hTERT-HPNE cells. CAP2 has also been shown to regulate the actin cytoskeleton^37^, which likely underlies the important roles in heart functions^38,39^. Considering that regulating the actin cytoskeleton is a key mechanism underlying the role of CAP1 in pancreatic cancer cell invasiveness, it is possible that CAP2 shares the role with CAP1 in regulating cancer cell invasiveness.

Our results support that the activity of CAP1, regulated through phosphorylation at S308/S310 tandem site, is important for the protein function in regulating the invasiveness of cancer cells. CAP1 phosphorylation is elevated in cancer cells, consistent with the reported hyper-activation of GSK3 in pancreatic cancer. Inhibition of GSK3 or re-expression of the phosphor mutants in the CAP1-knockdown PANC-1 cells, both disrupting transient phosphorylation of CAP1, consistently led to reduced cancer cell motility and invasion. The phosphor-mimetic DD mutant especially had severely compromised functions in rescuing the reduced motility and invasion in the CAP1-knockdown PANC-1 cells, and consistently, the mutant had defect in alleviating the enhanced stress fibers. Thus, these findings all point to a crucial role for S308/S310 phosphorylation in CAP1 function in cancer cell invasiveness. Interestingly, we found that growth factor PDGF induced CAP1 dephosphorylation at S308/S310 in both PANC-1 and AsPC-1 cancer cells, suggesting a likely role for CAP1 in mediating extracellular signals to control the invasiveness of pancreatic cancer cells.

It is somewhat intriguing that the more invasive cancer cells had elevated phosphorylation on CAP1 compared to that in the untransformed control cells, considering that dephosphorylated CAP1 is considered the ‘active’ form and transient phosphorylation is important for CAP1 function in regulating the actin cytoskeleton. We speculate that the presence of a potent phosphor-regulatory machinery (for which GSK3 is a part of) that cell can employ to drive transient phosphorylation of CAP1 and thus achieve spatially localized CAP1 activity may be the key, as this can facilitate localized actin dynamics essential for cell polarization and subsequent directional cell movement. It is possible that the increased pool of phosphorylated CAP1 in cancer cells reflects increased subcellular portions where CAP1 activity and local actin dynamics are reduced, which is a reasonable scenario to expect in a polarized cell. We attempted to stain phosphorylated population of CAP1 in the PANC-1 cells using the phosphor-specific antibody; however, pronounced non-specific binding of the antibody prevented yielding any conclusive results.

Recent studies have unraveled a cell context-dependent role for CAP1 in mammalian cell motility and cancer cell invasiveness^12–14,18^. Our findings so far suggest that the effect of CAP1 depletion on FAK activity is likely a key factor underlying the distinct, and often opposite, effects in different cell types. In HeLa and metastatic breast cancer cells, knockdown of CAP1 led to activation of FAK, and consistently, enhanced cell spreading and adhesion^12,18^. In contrast, knockdown of CAP1 in non-metastatic MCF-7 breast cancer cells actually led to reduced FAK activity and compromised cell adhesion^18^. As a result, in the CAP1-knockdown HeLa and the metastatic breast cancer cells, the positive effect from FAK activation is believed to have overcome the negative effect on cell motility and invasion due to the compromised actin filament turnover from loss of CAP1 function^11^. In pancreatic cancer cells, depletion of CAP1 led to reduced FAK activity, and it is likely that the reduced cancer cell invasiveness caused by CAP1 knockdown derives from decreases in both actin filament turnover and cell adhesion. Also, like in the non-metastatic breast cancer cells^18^, CAP1 knockdown in pancreatic cancer cells did not increase cell size. This is opposite to the case in HeLa and metastatic breast cancer cells, and the distinct effects on FAK activity are consistent with the effects on cell size, which is expected since activated FAK stimulates cell spreading. A question that remains to be answered is how depletion of CAP1 leads to activation of FAK in some cell types, whereas in others it exerts an opposite effect. We previously identified physical and functional interactions between CAP1 and FAK in HeLa cells^12^, and it would be interesting to determine if differential interaction between the two in different cells may underlie the different effects.

Unlike the case in breast cancer cells, we did not detect alterations in ERK activity in the CAP1-knockdown cells. Our results, including from the MTT proliferation assays, support that CAP1 does not play a significant role in regulating the proliferation of pancreatic cancer cells. The modest decrease in proliferation of the CAP1-knockdown stable PANC-1 cells was not statistically significant, which may derive from the reduced FAK activity since FAK is also involved in cell proliferation despite not being considered as a key regulator of cell proliferation^40^.

In summary, while we did not find evidence that supports considerable up-regulation of CAP1 in pancreatic cancer cells, we further establish a required role for CAP1 in not only the motility but also the invasion of pancreatic cancer cells. Moreover, we identify a crucial role for S308/S310 phosphorylation for CAP1 functions in regulating the actin cytoskeleton and invasiveness of pancreatic cancer cells. Our results support that transient phosphorylation at the regulatory site is required for the CAP1 functions in cancer cells. Furthermore, we demonstrate that CAP1 regulates FAK and cell adhesion, but not ERK or cancer cell proliferation. CAP1 is believed to promote pancreatic cancer cell invasiveness through both promoting actin filament turnover and controlling cell adhesion. Finally, our findings suggests a likely role for CAP1 in mediating extracellular growth factor signals, through phosphor-regulation at S308/S310, to control cancer cell invasiveness. Such novel mechanistic insights may ultimately lead to the development of strategies targeting CAP1 or its regulatory cell signals for improving the treatment outcome of pancreatic cancer.

## Materials and Methods

### Miscellaneous reagents for cell culture, transfection and Western blotting

The monoclonal antibody against human CAP1 has been previously described^41^. HGF and 6xHis antibody were from Sigma Aldrich (St. Louis, MO). Alexa-Flour 488 Phalloidin, fibronectin and PDGF were from Life Technologies Inc. (Carlsbad, CA). Mouse E-Cadherin antibody and Matrigel were from BD Biosciences (San Jose, CA). Antibodies against ERK1/2, phosphor-ERK1/2 (Thr202/Tyr204), FAK, phosphor-Tyr397 FAK and Vimentin were from Cell Signaling Technology Inc. (Danvers, MA). The GAPDH antibody was from Santa Cruz Biotechnology Inc. (Santa Cruz, CA). The polyclonal CAP2 antibody developed in our laboratory has been described previously^12,18^. The phosphor-specific antibody against S307/S309 on mouse CAP1 (S308/S310 on human CAP1) developed in our laboratory has been described previously^24^. Horseradish peroxidase-conjugated goat anti-rabbit and goat anti-mouse antibodies were from Jackson ImmunoResearch Laboratories Inc. (West Grove, PA). Western blotting was performed following standard immunoblotting techniques using enhanced chemiluminescence, by exposing an X-ray film for visualization. Western blot experiments were conducted for three times with similar results. Glass bottom tissue culture plates for fluorescent imaging were from MatTek Corp. (Ashland, MA) and FBS (Fetal Bovine Serum) was from Hyclone Laboratories Inc. (Logan, UT). FuGene 6 was from Promega (Madison, WI), and cells were transfected following the manufacturer’s instructions.

### Cell culture and treatment

All the cell lines were purchased from ATCC. PANC-1 cells were maintained in DMEM (Dulbecco’s Modified Eagle’s Medium) supplemented with 10% FBS. AsPC-1 cells were maintained in RPMI medium (ATCC) supplemented with 10% FBS. CFPAC cells were maintained in IMDM (Iscove’s modified Dulbecco’s medium, ATCC) supplemented with 10% FBS. Mia PaCa-2 cells were maintained in DMEM supplemented with 10% FBS and 2.5% of horse serum. The hTRET-HPNE cells were cultured in glucose-free DMEM supplemented with 25% M3 Base medium (INCELL; San Antonio, TX), 5% FBS, 10 ng/ml human recombinant Epidermal Growth Factor and 5.5 mM D-glucose and 750 ng/ml puromycin. All the cells were cultured at 37 °C, with 5% CO_2_ and the presence of penicillin (100 U/ml) and streptomycin (100 μg/ml). Cells were treated with 6′-bromo-3-(hydroxyimino)-[2,3′-biindolinylidene]-2′-one (BIO) (BioVision; Milpitas, CA), LiCl and PDGF at the indicated concentrations and time durations.

### Generation of CAP1 knockdown and re-expression stable cells

The shRNA constructs targeting human CAP1 used to establish stable CAP1 knockdown PANC-1 and AsPC-1 cells have been described^12,33^. Stable clones were established through selection with 1,500 μg/ml Neomycin for two weeks following transfection. For generating the stable knockdown pools, cells transfected with the shRNA and survived the subsequent Neomycin selection for three weeks were detached and collected as pools. To re-express WTCAP1 or the S307A/S309A (AA) and S307D/S309D (DD) phosphor mutants of mouse CAP1 in the CAP1-knockdown PANC-1 cells, constructs previously generated that harbor mismatches introduced to the S3 target sequence of mouse *CAP1* gene were transfected and selected^12,18,24^. Stable cells re-expressing CAP1 (WT, AA or DD) were generated by transfection of S3-3 cells, followed by selection with 300 μg/ml Zeocin for two weeks.

### Phase imaging and confocal immunofluorescence

Cells were observed and phase images were taken using a ZEISS Axiovert 200 M microscope with the IPLAB4 program. Cell size was measured using ImageJ similarly as we did previously^12,24^. For staining of the actin cytoskeleton, cells were grown on MatTek plates overnight, fixed with 3.7% paraformaldehyde for 30 min before permeabilization with PBS (phosphate-buffered saline) containing 0.5% Triton X-100. The samples were then incubated with Alexa-Fluor 488 Phalloidin for 1 hr, washed three times with PBS containing 0.1% Triton X-100 and mounted using Vectashield mounting medium from Vector Laboratories Inc. (Burlingame, CA). Confocal images were taken using a BD Pathway 855 Imaging Station with a 60x oil objective lenses, and stacks with a 0.5-μm increment were obtained.

### Cell migration, Matrigel invasion and MTT proliferation assays

Cell migration and invasion assays were conducted similarly as previously described^12,18,42^. For wound healing assays, cells were cultured overnight on 6-well plates until confluent. A scratch (wound) was then introduced and cells were further incubated for 16 hrs before images of the wound were captured. Cell motility in the wound healing assays was calculated following this formula: [cell motility = (wound width at 0 hr – wound width at n hr)/wound width at 0 hr]^18^. Transwell migration assays were conducted similarly as we previously did^12,18,42^. Sub-confluent cells were serum starved overnight and detached, and ~2 × 10^4^ cells were then plated in triplicate onto Transwell inserts with 8 µm size pores (Corning Inc.; Corning, NY), which had been placed in 12-well plates filled with media containing 10 µg/ml PDGF. The cells were incubated overnight, and non-migratory cells, which remained on the upper side of the insert, were removed with a cotton swab. The cells that had migrated to the opposite side of the membrane were stained and cells in four random fields excluding the edge areas were scored under a microscope. The data were analyzed using Student’s *t*-test. For invasion assays, the Transwell inserts pre-coated with Matrigel were used and the assays were conducted following similar procedures to those in the Transwell migration assays otherwise. The MTT proliferation assays were conducted similarly as we used previously^18^.

## Supplementary information

Supplementary Info

## References

[1] Hanahan D, Weinberg RA (2000). The hallmarks of cancer. Cell.

[2] Maitra A, Hruban RH (2008). Pancreatic cancer. Annu Rev Pathol.

[3] Hall A (2009). The cytoskeleton and cancer. Cancer metastasis reviews.

[4] Fife CM, McCarroll JA, Kavallaris M (2014). Movers and shakers: cell cytoskeleton in cancer metastasis. Br J Pharmacol.

[5] Fedor-Chaiken M, Deschenes RJ, Broach JR (1990). *SRV2*, a gene required for RAS activation of adenylate cyclase in yeast. Cell.

[6] Field J (1990). Cloning and characterization of *CAP*, the *S*. *cerevisiae* gene encoding the 70 kd adenylyl cyclase-associated protein. Cell.

[7] Ono S (2013). The role of cyclase-associated protein in regulating actin filament dynamics - more than a monomer-sequestration factor. J Cell Sci.

[8] Hubberstey AV, Mottillo EP (2002). Cyclase-associated proteins: CAPacity for linking signal transduction and actin polymerization. The Faseb J.

[9] Moriyama K, Yahara I (2002). Human CAP1 is a key factor in the recycling of cofilin and actin for rapid actin turnover. J Cell Sci.

[10] Yu G, Swiston J, Young D (1994). Comparison of human CAP and CAP2, homologs of the yeast adenylyl cyclase-associated proteins. J Cell Sci.

[11] Zhou GL, Zhang H, Field J (2014). Mammalian CAP (Cyclase-associated protein) in the world of cell migration: Roles in actin filament dynamics and beyond. Cell Adh & Migr.

[12] Zhang H (2013). Mammalian adenylyl cyclase-associated protein 1 (CAP1) regulates cofilin function, the actin cytoskeleton, and cell adhesion. J Biol Chem.

[13] Bertling E (2004). Cyclase-associated protein 1 (CAP1) promotes cofilin-induced actin dynamics in mammalian nonmuscle cells. Mol Biol Cell.

[14] Yamazaki K (2009). Adenylate cyclase-associated protein 1 overexpressed in pancreatic cancers is involved in cancer cell motility. Lab Investigation.

[15] Liu Y (2013). Upregulated expression of CAP1 is associated with tumor migration and metastasis in hepatocellular carcinoma. Pathol Res Pract.

[16] Tan M (2013). Overexpression of adenylate cyclase-associated protein 1 is associated with metastasis of lung cancer. Oncol Rep.

[17] Li M (2013). Downregulated expression of the cyclase-associated protein 1 (CAP1) reduces migration in esophageal squamous cell carcinoma. Jpn J Clin Oncol.

[18] Zhang H, Zhou GL (2016). CAP1 (Cyclase-Associated Protein 1) Exerts Distinct Functions in the Proliferation and Metastatic Potential of Breast Cancer Cells Mediated by ERK. Sci Rep.

[19] Yu XF (2014). Knocking down the expression of adenylate cyclase-associated protein 1 inhibits the proliferation and migration of breast cancer cells. Exp Mol Pathol.

[20] Huttenlocher A, Sandborg RR, Horwitz AF (1995). Adhesion in cell migration. Curr Opin Cell Biol.

[21] Tilghman RW (2005). Focal adhesion kinase is required for the spatial organization of the leading edge in migrating cells. J Cell Sci.

[22] Subauste MC (2004). Vinculin modulation of paxillin-FAK interactions regulates ERK to control survival and motility. J Cell Biol.

[23] Strippoli R (2015). Caveolin-1 deficiency induces a MEK-ERK1/2-Snail-1-dependent epithelial-mesenchymal transition and fibrosis during peritoneal dialysis. EMBO Mol Med.

[24] Zhou GL, Zhang H, Wu H, Ghai P, Field J (2014). Phosphorylation of the cytoskeletal protein CAP1 controls its association with cofilin and actin. J Cell Sci.

[25] Zhang JS (2011). Mutant K-Ras increases GSK-3beta gene expression via an ETS-p300 transcriptional complex in pancreatic cancer. Oncogene.

[26] Lieber M, Mazzetta J, Nelson-Rees W, Kaplan M, Todaro G (1975). Establishment of a continuous tumor-cell line (panc-1) from a human carcinoma of the exocrine pancreas. Int J Cancer.

[27] Schoumacher RA (1990). A cystic fibrosis pancreatic adenocarcinoma cell line. Proc Natl Acad Sci USA.

[28] Chen WH (1982). Human pancreatic adenocarcinoma: *in vitro* and *in vivo* morphology of a new tumor line established from ascites. In vitro.

[29] Yunis AA, Arimura GK, Russin DJ (1977). Human pancreatic carcinoma (MIA PaCa-2) in continuous culture: sensitivity to asparaginase. Int J Cancer.

[30] Lee KM, Nguyen C, Ulrich AB, Pour PM, Ouellette MM (2003). Immortalization with telomerase of the Nestin-positive cells of the human pancreas. Biochem Biophys Res Commun.

[31] Meijer L, Flajolet M, Greengard P (2004). Pharmacological inhibitors of glycogen synthase kinase 3. Trends Pharmacol Sci.

[32] Zhang F, Phiel CJ, Spece L, Gurvich N, Klein PS (2003). Inhibitory phosphorylation of glycogen synthase kinase-3 (GSK-3) in response to lithium. Evidence for autoregulation of GSK-3. J Biol Chem.

[33] Wang C, Zhou GL, Vedantam S, Li P, Field J (2008). Mitochondrial shuttling of CAP1 promotes actin- and cofilin-dependent apoptosis. J Cell Sci.

[34] Sun T, Rodriguez M, Kim L (2009). Glycogen synthase kinase 3 in the world of cell migration. Dev Growth Differ.

[35] Woodgett JR (2001). Judging a protein by more than its name: GSK-3. Sci STKE.

[36] Xiang, C., Chen, J. & Fu, P. HGF/Met Signaling in Cancer Invasion: The Impact on Cytoskeleton Remodeling. *Cancers (Basel)* **9** (2017). 10.3390/cancers9050044 PMC5447954 28475121

[37] Kosmas K (2015). CAP2 is a regulator of the actin cytoskeleton and its absence changes infiltration of inflammatory cells and contraction of wounds. Europ J Cell Biol.

[38] Peche VS (2013). Ablation of cyclase-associated protein 2 (CAP2) leads to cardiomyopathy. Cell Mol Life Sci: CMLS.

[39] Field J (2015). CAP2 in cardiac conduction, sudden cardiac death and eye development. Sci Rep.

[40] Gilmore AP, Romer LH (1996). Inhibition of focal adhesion kinase (FAK) signaling in focal adhesions decreases cell motility and proliferation. Mol Biol Cell.

[41] Freeman NL, Field J (2000). Mammalian homolog of the yeast cyclase associated protein, CAP/Srv2p, regulates actin filament assembly. Cell Motil Cytoskeleton.

[42] Zhou GL (2006). Opposing roles for Akt1 and Akt2 in Rac/Pak signaling and cell migration. J Biol Chem.

