# Supplementary material for: Phosphorylation Regulates CAP1 (Cyclase-Associated Protein 1) Functions in the Motility and Invasion of Pancreatic Cancer Cells
Source: Sci Rep. 2019 Mar 20;9:4925. doi: 10.1038/s41598-019-41346-3 (PMC6426867; doi:10.1038/s41598-019-41346-3)
Supplement: Supplementary file 1 — Supplementary Info [file 41598_2019_41346_MOESM1_ESM.pdf]

Title Page for Supplementary Information

Phosphorylation Regulates CAP1 (Cyclase-Associated Protein 1) Functions in the Motility and Invasion of Pancreatic Cancer Cells

Huhehasi Wu[1#], Rokib Hasan[2#], Haitao Zhang[2], Joshua Gray[1], Dominic Williams[1], Morgan Miller[1], Faith Allen[1], Virlan Lee[1], Jennifer Y. Xie[3], Thomas Kelly[4,5] & Guo-Lei Zhou[1,2*]

1. Department of Biological Sciences, Arkansas State University
2. Molecular Biosciences Graduate Program, Arkansas State University
3. Department of Basic Sciences, New York Institute of Technology College of Osteopathic Medicine at Arkansas State University
4. Department of Pathology, University of Arkansas for Medical Sciences
5. Winthrop P. Rockefeller Cancer Institute, University of Arkansas for Medical Sciences

*****: To whom correspondence should be addressed: gzhou@astate.edu; Tel: 870-680-8588; Department of Biological Sciences, Arkansas State University; P.O. Box 599, State University, AR 72467

#: These authors contributed equally to this work.

Supplementary Figure 1

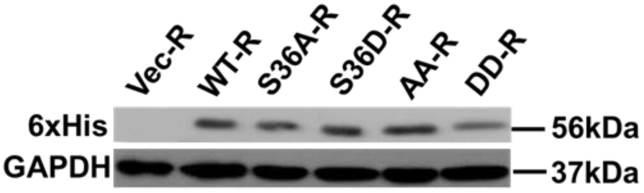

Supplementary Figure 1: Confirmation of re-expression of WTCAP1 and the AA and DD phosphor mutants, along with other two phosphor-mutants S36A and S36D in the CAP1-knockdown S3-3 stable PANC-1 cells. The expression was confirmed in Western blotting using an antibody against the 6xHis tag of the re-expressed protein. Two irrelevant lanes (with samples of S36A and S36D) were removed from this Western blot image, and presented in Fig. 4A.

Supplementary Figure 2

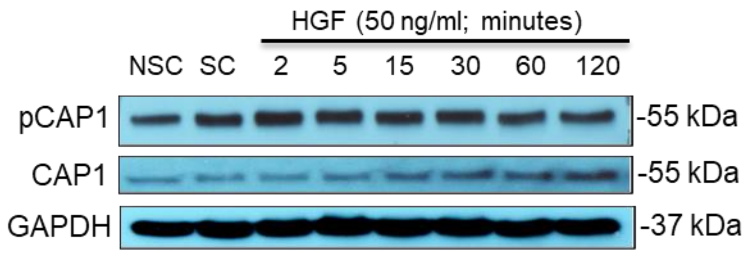

Supplementary Figure 2: Hepatocyte Growth Factor (HGF) did not have an obvious effect in inducing CAP1 dephosphorylation. PANC-1 cells were serum-starved for 22 hrs and treated with 50ng/ml HGF for the indicated durations, and phosphorylation at S307/S309 on CAP1 was detected in Western blotting. CAP1 and GAPDH were also detected, with the latter serving as a loading control. Non-starved control cells (NSC) and starved control cells (SC), which were serum-starved but without HGF stimulation, were also included.

Supplementary Figure 3

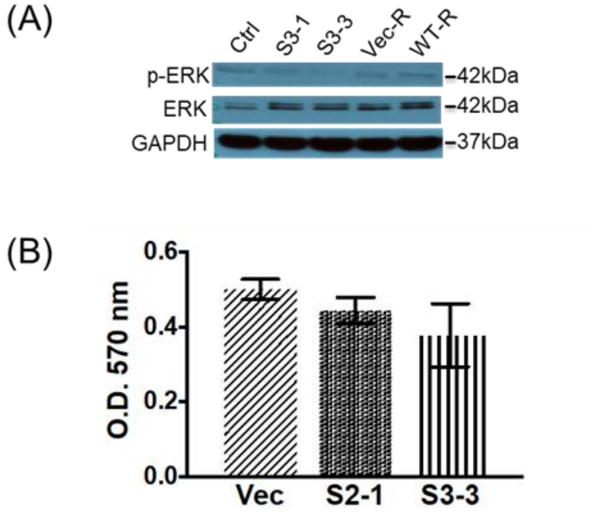

Supplementary Figure 3: Depletion of CAP1 in PANC-1 cells did not cause significant alterations in ERK or cell proliferation. **(A)** Knockdown of CAP1 in PANC-1 cells, or re-expression of CAP1 in the knockdown cells, did not cause remarkable changes in ERK activity. The control cells and two knockdown stable clones (S3-1 and S3-3), along with S3-3 cells harboring an empty re-expression vector (Vec-R) or re-expressing WTCAP1 (WT-R) were used in testing ERK expression and phosphorylation. **(B)** MTT assays show that the CAP1-knockdown cells that derive from both S2 and S3 shRNA constructs did not have significantly reduced proliferation. The p values comparing the control (Vec) cells to S2-1 cells was at 0.209, and that between control cells and S3-3 cells was at 0.223, respectively.
